# Study on the Effect of Wing Bud Chitin Metabolism and Its Developmental Network Genes in the Brown Planthopper, *Nilaparvata lugens*, by Knockdown of *TRE* Gene

**DOI:** 10.3389/fphys.2017.00750

**Published:** 2017-09-26

**Authors:** Lu Zhang, Ling-Yu Qiu, Hui-Li Yang, Hui-Juan Wang, Min Zhou, Shi-Gui Wang, Bin Tang

**Affiliations:** Hangzhou Key Laboratory of Animal Adaptation and Evolution, College of Life and Environmental Sciences, Hangzhou Normal University, Hangzhou, China

**Keywords:** *Nilaparvata lugens*, trehalase, wing bud, RNA interference, chitin synthase, chitinase

## Abstract

The brown planthopper, *Nilaparvata lugens* is one of the most serious pests of rice, and there is so far no effective way to manage this pest. However, RNA interference not only can be used to study gene function, but also provide potential opportunities for novel pest management. The development of wing plays a key role in insect physiological activities and mainly involves chitin. Hence, the regulating role of trehalase (TRE) genes on wing bud formation has been studied by RNAi. In this paper, the activity levels of TRE and the contents of the two sugars trehalose and glucose were negatively correlated indicating the potential role of TRE in the molting process. In addition, *NlTRE1-1* and *NlTRE2* were expressed at higher levels in wing bud tissue than in other tissues, and abnormal molting and wing deformity or curling were noted 48 h after the insect was injected with any double-stranded TRE (*dsTRE*), even though different TREs have compensatory functions. The expression levels of *NlCHS1b, NlCht1, NlCht2, NlCht6, NlCht7, NlCht8, NlCht10, NlIDGF*, and *NlENGase* decreased significantly 48 h after the insect was injected with a mixture of three kinds of *dsTREs*. Similarly, the TRE inhibitor validamycin can inhibit *NlCHS1* and *NlCht* gene expression. However, the wing deformity was the result of the *NlIDGF, NlENGase, NlAP*, and *NlTSH* genes being inhibited when a single *dsTRE* was injected. These results demonstrate that silencing of *TRE* gene expression can lead to wing deformities due to the down-regulation of the *AP* and *TSH* genes involved in wing development and that the TRE inhibitor validamycin can co-regulate chitin metabolism and the expression of wing development-related genes in wing bud tissue. The results provide a new approach for the prevention and management of *N. lugens*.

## Introduction

Trehalose is widely distributed in insect tissues (including the epidermis and gut) and is the extracellular source of sugar for many insect species (Becker et al., 1996). Trehalose is known as the blood sugar of insects and plays a key role in various physiological processes (Kikuta et al., 2012; Yasugi et al., 2017). Trehalase (TRE), in insects, is divided into two types- soluble TRE, also called TRE1 or Treh1, and membrane-bound TRE, also called TRE2 or Treh2 (Shukla et al., 2015; Tang et al., 2016; Zhao et al., 2016). TRE1 is involved in the hydrolysis of endogenous trehalose, and TRE2 functions in the assimilation of exogenous trehalose as a carbon source (Becker et al., 1996; Chen et al., 2010a). Most insect species, including *Spodoptera exigua* (Tang et al., 2008; Chen et al., 2010a), *Apis mellifera* (Lee et al., 2007), *Bombyx mori* (Mitsumasu et al., 2005; Kamei et al., 2011), *Laodelphax striatellus* (Zhang et al., 2012), *Omphisa fuscidentalis* (Tatun et al., 2008a,b), and *Bemisia tabaci* (Wang et al., 2014) have one soluble TRE gene as well as one membrane-bound TRE gene (Bansal et al., 2013). In addition, some insect have more than one soluble TRE; two Treh1 were found in *Leptinotarsa decemlineata* (Shi J. F. et al., 2016), five Treh1 were found in *Harmonia axyridis* (Tang et al., 2014; Shi Z. K. et al., 2016) and four Treh1 were found in *Tribolium castaneum* (Tang et al., 2016). In addition, two TRE1 and one TRE2 have been cloned and reported in *Nilaparvata lugens* (Zhao et al., 2016). All of the different TREs can regulate and maintain trehalose balance during insect development and physiological processes.

Chitin is a major component of the exoskeleton, which includes the cuticle, leg, wing bud, feelers, and insect outside structure; it is also found in the internal structures of many insects and other arthropods, including the peritrophic matrix, inner cuticular linings of the alimentary canal, tracheal system, genital ducts, and ducts of the various dermal glands (Zhu et al., 2016). During insect development and metamorphosis, the chitin contents of the cuticle fluctuate as a complex function of the activities of chitin synthase A (CHS-A), which forms chitin via the chitin synthesis pathway; chitinase and chitinase-like (Cht), which degrade chitin to low-molecular-weight chitooligosaccharides; and β*-N-*acetylglucosaminidases (NAGs), which catalyze the stepwise removal of terminal, non-reducing β-*N*-acetylglucosamine residues from chitooligosaccharides (Zhu et al., 2016). In addition, that the chitin biosynthesis pathway is a well-established key target for pest control, and *TRE* is the first gene in this pathway (Tang et al., 2017). Previous studies have shown that target gene silencing via RNA interference (RNAi) or inhibition of TRE activities can lead to insect death or morphological defects because TRE plays an important regulatory role in chitin metabolism (Zhao et al., 2016; Tang et al., 2017). TRE regulates the expression of CHS and Cht in the insect cuticle and midgut, and inhibiting chitin biosynthesis kills pests when *TRE* gene expression is suppressed or knocked down (Chen et al., 2010a; Zhang et al., 2012; Tang et al., 2016, 2017; Zhao et al., 2016). When insect chitin synthesis or degradation malfunction or the chitin content are decreases, normal molting does not occur, and abnormalities are observed (Zhao et al., 2016; Zhang et al., 2017a).

When insects undergo molting, the cuticle of the exoskeleton is renewed by degrading the old chitin and cuticle proteins and synthesizing new ones (Deng et al., 2016). The wing development network includes many genes, of which *EN* (engrailed), *TSH* (teashirt), *WG* (wingless), *DLL* (distal-less), *VG* (vestigial), *SC* (scute), *VVL* (ventral veins lacking), *CI* (cubitus interruptus), and *AP* (apterous) play important roles (Xue et al., 2013, 2014). Proper development of the wing in insects involves the coordinate action of several genes (Ramesh Kumar et al., 2016). There are three key regulatory genes involved in insect wing development: *WG, VG*, and *AP*; these genes were studied in two basalinsects (Niwa et al., 2010). Wing deformity was observed when double-stranded (ds) VG, dsAP, dsCI, and dsUBX (Ultrabithorax) were injected into *N. lugens* (Xue et al., 2014); wing deformity was also observed when a high level of validamycin was injected due to decreased expression of the *EN, WG, SC, VVL, CI*, and *AP* genes (Tang et al., 2017).

The selector gene *AP* plays a key role in the development of the *Drosophila melanogaster* wing because it governs the establishment of the dorsal-ventral (DV) compartment boundary (Bieli et al., 2015). Repression of *TSH* is important for the establishment of distal wing fate, as ectopic expression of *TSH* blocks *WG* expression at the DV boundary and interferes with wing pouch development (Casares and Mann, 2000; Wu and Cohen, 2002). The homeobox gene *DLL* is a crucial transcription factor involved in limb development and is highly conserved among both vertebrates and invertebrates (Sunwoo et al., 2008; Pechmann et al., 2011; Plavicki et al., 2012). Different AP alleles can lead to a wide range of wing phenotypes (Stevens and Bryant, 1985), while loss of *TSH* gene expression can result in the fusion of the proximal structures of the wing and halteres to the thoracic cuticle (Soanes et al., 2001). Wings that are ~39.5% curled were observed when the *NlDLL* gene was knockdown by the way of RNAi; RNAi could also lead to an abnormal phenotype in not only the wing, which was extremely small, but also the haltere, which was small and disrupted, and produce defects, for example, extraordinary bristles (Lin et al., 2014).

Rice is the most important cereal crop in the Asia-Pacific region. The brown planthopper (BPH), *N. lugens* (Stål), a long-distance migration insect with wing dimorphism, is the most destructive rice pest in Asia. Macropterous adults have the ability to fly long distances and invade rice-growing areas, while brachypterous adults are adapted for reproduction and can produce numerous offspring in rice fields (Xue et al., 2013). RNAi has been used as a gene silencing strategy via the introduction of long dsRNA for pest control (Liu et al., 2010, 2015; Wang et al., 2012, 2013; Zhao et al., 2016; Li et al., 2017). In previous studies, we found that *N. lugens* could not complete the molting process and observed an abnormal phenotype when the expression of the three *TRE* genes was knocked down by RNAi (Zhao et al., 2016) or when TRE activities were inhibited by the TRE inhibitor validamycin (Tang et al., 2017). And it is known that TRE participate and regulates both homeostasis and development (Thompson, 2003; Tatun et al., 2008a,b; Shukla et al., 2015). Abnormal development, weight loss, reduced chitin synthesis, decreased chitin content, hampered growth, decreased flight capacity, and lethal metamorphosis are found when TRE activity is inhibited (Shukla et al., 2015; Shi J. F. et al., 2016; Tang et al., 2017; Zhang et al., 2017b). These results indicated that insect chitin synthesis or degradation was severely affected and that insect chitin decreased when *TRE* gene expression or protein activities were inhibited (Zhang et al., 2017b). From the previous studies, *TRE* genes play a key role in the wing development and by the way of wing-related genes expression. However, it is not clear how TRE regulates wing development. Therefore, the present study aimed to further investigate the functions of *TRE* and validamycin in *N. lugens* wing development by evaluating gene expression patterns when different *TRE* genes or TRE activities are inhibited. In other words, the potential mechanism was elucidated for trehalase as an effective target for the control of *N. lugens*.

## Materials and methods

### Insects

The BPHs used in this study were from the China National Rice Research Institute (Hangzhou, China), and all rice cultivars were infected with TN1 (Taichung Native 1). The experimental insects were reared on fresh TN1 rice in an artificial climate chamber or in an artificial climate chamber at 25 ± 1°C, (70 ± 5)% relative humidity, and 14L:10D photoperiod. All experiments were performed under the same conditions. At each molt, developmental stages were synchronized by collecting new nymph, pupae, or adults. The insects used for the microinjection of RNAi were BPHs on the first day of the 5th instar nymph.

### Trehalase activities detected during different developmental stages

An experimental population was used in this trial. Insects were subjected to soluble and membrane-bound TRE activity analyses at different stages from the beginning of the 5th instar until 72 h of the adults. In this experiment, several insects' whole body (about 5–10 insects) at the same developmental stage were used to detect TRE activity, trehalose, and glycogen contents every 12 h and was replicated three times.

TRE activity assays were conducted according to the earlier methods with some modifications (Tatun et al., 2008a,b). Briefly, insects were homogenized in phosphate buffer (pH 7.0; Sangon Biotech, China) and the homogenate was centrifuged at 1,000 g for 20 min at 4°C. Subsequently, 350 μl of the supernatant was removed and ultracentrifuged at 20,800 g for 60 min at 4°C. The remaining supernatant was used to determine the protein, trehalose, and glycogen concentrations as described below. The supernatant obtained from ultracentrifugation was used to determine TRE1 activity, while the sediment was re-suspended in phosphate buffer (pH 7.0) and used to evaluate TRE2 activity. The protein and glucose concentrations in both the supernatant and sediment fractions were determined. To investigate TRE activity, the supernatantor suspension obtained from ultracentrifugation (60 μl) was uniformly mixed with 75 μl of 40 mM trehalose (Sigma-Aldrich, USA) and 165 μl of phosphate buffer (pH 7.0). The mixture was then incubated at 37°C for 60 min and centrifuged at 12,000 g for 10 min at 4°C. The resulting supernatants (50 μl) were used to measure TRE activity using the Glucose (Go) Assay Kit (Sigma-Aldrich) according to the manufacturer's instructions. The protein concentration was determined using the BCA Protein Assay Kit (Beyotime, China) according to the manufacturer's instruction.

### Trehalose and glycogen assays during different developmental stages

The trehalose content assay was conducted according to the earlier method with some modifications (Leyva et al., 2008). The trehalose content was measured in 10 μl of the supernatant described above using anthrone. The reagent was prepared by dissolving 0.1 g of anthrone (Sinopharm, China) in 100 ml of concentrated sulfuricacid (98%; Sinopharm), kept protected from light, and used within 12 h. To measure the trehalose content, 10 μl of 1% sulfuric acid was added to 10 μl of sample and mixed uniformly. The obtained mixture was incubated at 90°C for 10 min and then cooled at 0°C for 3 min. In the second step, 10 μl of 30% potassium hydroxide (Sinopharm) solution was added to the sample and mixed uniformly. The obtained mixture was incubated at 90°C for 10 min and then cooled at 0°C for 3 min. In the final step, 200 μl of anthrone reagent was added to the sample and mixed uniformly. The obtained mixture was incubated at 90°C for 10 min and then cooled at 0°C, and the absorbance was read at 620 nm. To determine the glycogen concentration, another 10-μl aliquot of the supernatant described in the previous section was incubated at 40°C for 4 h in the presence of starch glycosidase enzymes (Sigma-Aldrich) to hydrolyze glycogen into glucose. The glucose content was determined using the glucose (Go) Assay Kit according to the manufacturer's instructions.

### Total RNA isolation and expression studies

In the experiment of TRE genes expression pattern, the different tissues including head, leg, wing bud, cuticle, and fat body, from about 30 to 50 individuals were used. Three replicate samples of all the tissues were used to extract total RNA by using TRIzol (Invitrogen, Carlsbad, California, USA), respectively. The expression of three *TRE* genes in the head, leg, wing bud, cuticle, and fat body of *N. lugens* were detected by qRT-PCR.

In the RNAi experiments, total RNA was extracted from the wing bud of *N. lugens* using TRIzol (Invitrogen, Carlsbad, California, USA) following the manufacturer's instructions. The total RNA integrity was determined by 1% agarose gels electrophoresis, and the RNA concentration and purity were measured using a Nanodrop 2000 spectrophotometer (Thermo Fisher Scientific, Waltham, MA, USA). First-strand cDNA synthesis was performed using the Prime Script®RT reagent Kit With gDNA Eraser (Takara, Kyoto, Japan) and stored at −20°C.

Complementary DNA synthesis and quantitative real-time polymerase chain reaction (qRT-PCR) were performed to analyze the distribution of *NlTRE1-1, NlTRE1-2*, and *NlTRE2* using gene-specific primers (Table 1). Using 1 mg of total RNA as a template and a specifically designed Nl-18S primer pair (Table 1), the stability of 18S RNA was demonstrated by PCR performed under the following conditions: 95°C for 5 min, 28 cycles at 95°C for 30 s, 60°C for 30 s, and 72°C for 30 s, followed by a final extension at 72°C for 10 min. The relative mRNA expression levels of unique genes were assessed via qRT-PCR with a SYBR Green master mix (SYBR Green Premix Ex Taq, Takara, Japan) in a Bio-Rad CFX96TM Real-Time PCR Detection System (Bio-Rad Laboratories Inc., Hercules, CA, USA). Production of the standard and melting curves confirmed the specificity and accuracy of the primers. All primers were designed to determine the expression levels of the corresponding homologous genes of the trehalose and chitin metabolism pathways (Zhang et al., 2011; Tang et al., 2017), including the following: three *TREs, CHS1* and its transcripts *CHS1a* and *CHS1b* (Wang et al., 2012), 10 *Chts*, endo-β-N-acetylglucosaminidase (*ENGase*), and imaginal disc growth factor (*IDGF*) (Table 3, Xi et al., 2015b). Additionally, the relative expression levels of wing development network-related genes were detected via qRT-PCR, including the following: *EN, TSH, WG, DLL, VG, SC, VVL, CI*, and *AP* (Table 4, Tang et al., 2017).

**Table 1:** The primers of three trehalase and *18S* gene for qRT-PCR.

**Gene**	**GenBank number**	**Forward (5′–3′)**	**Reverse (5′–3′)**	**Length of target fragment (bp)**
*NlTRE1-1*	FJ790319	GCCATTGTGGACAGGGTG	CGGTATGAACGAATAGAGCC	132
*NlTRE1-2*	KU556829	GATCGCACGGATGTTTA	AATGGCGTTCAAGTCAA	178
*NlTRE2*	GQ397451	TCACGGTTGTCCAAGTCT	TGTTTCGTTTCGGCTGT	197
*Nl-18S*	–	CGCTACTACCGATTGAA	GGAAACCTTGTTACGACTT	165

Each PCR was performed in a 20-μl volume, including 1 μl of each primer, 10 μl of SYBR buffer, 7 μl of ultrapure water, and 1 μl of cDNA. The qRT-PCR experiment was performed according to this cycling regime: preincubation at 95°C for 3 min, 35 cycles of 95°C for 10 min and 60°C for 30 s. The amplification of *18S RNA* was quantified as an internal control. After amplification, a melting curve analysis was performed in triplicate, and the results were averaged. The values were calculated using three independent biological samples, and the 2^−ΔΔCT^ method was used for the analysis of relative gene expression (Livak and Schmittgen, 2001).

### dsRNA synthesis and injections

Using *N. lugen*s cDNA template and specific primers containing the T7 promoter sequence at their 5' ends (Table 2), regions of three *NlTRE* genes were amplified by RT-PCR. The profile used in the reactions included 40 cycles of 95°C for 30 s, 58°C for 30 s, and 72°C for 45 s and a final extension at 72°C for 10 min. Purified *TRE* amplicons were transcribed *in vitro* to synthesize dsRNA using the T7 RiboMAX Express RNAi System (Promega Corporation, Madison, USA) (Zhao et al., 2016). A green fluorescence protein (*GFP*) amplicon was used as the control. Sense and anti-sense strands were first produced in two separate transcription procedures and then mixed for annealing. Reactions were incubated for 10 min at 70°C and then placed on an ice bath for 20 min. Finally, dsRNAs were precipitated with 95% ethanol and 3 M sodium acetate (pH 5.2), washed with 70% ethanol, air dried, and resuspended. The integrity and quantity of dsRNAs were evaluated by spectroscopy with Nanodrop 2000 (Thermo Fisher Scientific) and by agarose gel electrophoresis.

**Table 2:** Primers used for double stranded RNA synthesis.

**Primer name**	**Primer sequence (5′–3′)**
DSNl*TRE1-1*-F	GATGCAATCAAGGAGGTGTTATGGC
DSNl*TRE1-1*-R	CGTATTCACCTCCACCTCCGT
DSNl*TRE1-1*-FT	T7-GATGCAATCAAGGAGGTGTTATGGC
DSNl*TRE1-1*-RT	T7-CGTATTCACCTCCACCTCCGT
DSNl*TRE1-2*-F	AGATGAAGGCATGTGGTTCG
DSNl*TRE1-2*-R	CATCGATTCGCCAACTGGTAAGC
DSNl*TRE1-2*-FT	T7-AGATGAAGGCATGTGGTTCG
DSNl*TRE1-2*-RT	T7-CATCGATTCGCCAACTGGTAAGC
DSNl*TRE2*-F	CCAACTGCTATGACACCGACAAG
DSNl*TRE2*-R	GGGTTCAGATCCTGCCGTCGCT
DSNl*TRE2*-FT	T7-CCAACTGCTATGACACCGACAAG
DSNl*TRE2*-RT	T7-GGGTTCAGATCCTGCCGTCGCT
DSNl*GFP*-F	AAGGGCGAGGAGCTGTTCACCG
DSNl*GFP*-R	CAGCAGGACCATGTGATCGCGC
DSNl*GFP*-FT	T7-AAGGGCGAGGAGCTGTTCACCG
DSNl*GFP*-RT	T7-CAGCAGGACCATGTGATCGCGC

*T7 sequence: GGATCCTAATACGACTCACTATAGG*.

**Table 3:** Primers used for qRT-PCR measurements of chitin metabolism genes.

**Gene**	**GenBank number**	**Forward (5′–3′)**	**Reverse (5′–3′)**	**Length of target fragment (bp)**
*NlCHS1*	AEL88648	CCGCAAACGATTCCTACAGA	AGGTCCTTGACGCTCATTCC	222
*NlCHS1a*	JQ040014	TGTTCTTGCTACAACTCAATAAA	ACACCAATCCGATAGGCTC	141
*NlCHSb*	JQ040013	GCTGTCTTTGCTTTCTTCAT	ACACCAATCCGATAGGCTC	187
*NlCht1*	AJO25036	AGGTGGTTAGGGACGAGGAG	TGCGCTTGACATAGTTGGACT	114
*NlCht2*	AJO25037	GCAGATTTCTGGACAGGGAA	TGACGCACAAGCGGGAAG	226
*NlCht3*	AJO25038	CTACACCTCTGGCTAAACTCGG	AACTTGTCCTTGCGGCTGAT	235
*NlCht4*	AJO25039	TTGAGGAGGTTCACGGGTCT	CCTTACTGGAAACGAGGTTGG	112
*NlCht5*	AJO25040	AAAGCGTTCGTGATGAAATAGC	GATCCTTTGCCTCAATCCAAT	183
*NlCht6*	AJO25041	GCTGGTAAGGAGATGCTATTCG	GTGGTTCTAAGGCTGGCTGTC	155
*NlCht7*	AJO25042	CTACTCTGCCATCCCATTCCT	GTCTGGGTTTCTTCACTTCCTG	161
*NlCht8*	AJO25043	GAACAAAGTGCAAACTCAGTC	C CACCTTCTGTGGCTTCTGG	106
*NlCht9*	AJO25044	GTGCGGTATTGGTTGAAGAGG	GGTATAACGTGATTCCGAGCC	147
*NlCht10*	AJO25045	CAAGCCAATACCCAACAAAC	ACAGCAAATCCATAGAGCACA	177
*IDGF*	AJO25056	AAAAGAACGAGGAGGAGGG	TTGCTTGAGGATGGGGTAC	170
*ENGase*	AJO25057	TGTGGCAAGACTTCGTTA	ATGGGAGGGTTGGGATAG	282

**Table 4:** Primers used for qRT-PCR measurements of wing development network genes.

**Gene**	**BPH ID**	**Forward (5′–3′)**	**Reverse(5′–3′)**
*NlEN*	NLU020829.1	CTCACCTCCAGAAATGC	CTTCAGGCGAGACAGC
*NlTSH*	NLU025765.1	TGGTAGGAGGAGACAAA	TTCCAGCAGAATGGAGT
*NlWG*	NLU010606.1	ATAACCTGCTACCCTTGTCA	GTTCCACCTCCTGTTTCTG
*NlDLL*	NLU020890.1	CGTACCACCCTTACCAGC	TTGCCTTTGCCATTGTT
*NlVVL*	NLU013430.1	GCTCCTGTCGTCTCCTCA	CGGGTGTTGGTGGTTGT
*NlCI*	NLU023528.1	ACGGGAGGTGGTGGATT	CTGACATTGGAGTCGCTGA
*NlVG*	NLU025618.1	AGCAACTACCAGAGCACCAA	AGCAACAGGCTGCCATAC
*NlSC*	NLU020108.1	GCAAGCGGAGAATCAGTTT	GTCACCGACACGGGGATG
*NlAP*	NLU018087.2	TGGAGGTGGCGTTTGGC	GCGTCAGGGTTATGGTTG

Using an IM-31 microinjector (NARISHIGE, JAPAN), *dsTRE1-1, dsTRE1-2, dsTRE2*, mixture of *dsTRE1-1* and *dsTRE1-2* mixture, and mixture of three *dsTREs* (200 ng of each) were injected into the abdomen of the *N. lugens* nymph. Control groups were injected with *dsGFP* or with 100 nl (10 μg/μl and 1 μg of each insect) of validamycin. Validamycin (C178990, Lot: 20306) is a specific trehalase inhibitor. It was obtained from Dr. Ehrenxtorfer and prepared in Germany. The efficiency of gene knockdown resulting from RNAi was calculated as the ratio of gene expression between insects injected with target dsRNAs and GFP dsRNA, determined at 48 h after injection.

### Sample collection, phenotype observations, and photography

DsRNA was injected into fifth-instar larvae of *N. lugens*, every 12 h after dsRNA injections, dead individuals (assumed to be caused by physical damage due to injection) were identified and discarded. The remaining treated insects were grouped into three replicates (exceed 30 nymphs each group). In addition, the wing tissues of other abnormal insects were collected 48 h after RNAi treatment for subsequent analysis of the expression of TRE, chitin metabolism-, and wing development-related genes by qRT-PCR. Photographs were taken of abnormal insects, especially those with deformed wing development, as well as of insects in the different dsRNA injection treatments.

### Statistical analyses

The statistical significance of differences in trehalase activities, trehalose, and glycogen contents were determined by one-way analysis of variance (ANOVA) and analyzed by Tukey's test. Comparisons of different developmental were made with a two-way (ANOVA) followed by Tukey's test. The significance level was set at α = 0.05.

As controls, the mRNA expression levels were designated in the non-injected and dsGFP-injected groups. In this study, all data obtained were analyzed through one-way analysis of variance (ANOVA) and presented as the means ± standard errors (SEs) of 3–6 biological replicates. In Duncan's new multiple range test, a *P*-value below 0.01 or 0.05 was considered extremely significant or significant, respectively. A double asterisk indicates a highly significant difference in mRNA levels between each of the dsNlTRE-injected groups and the dsGFP group measured at the same time (*P* < 0.01, *T*-test), and an asterisk or different capital letter indicates a significant difference (*P* < 0.05, *T*-test).

## Results

### Analysis of trehalase activity and the concentrations of trehalose and glycogen during the different developmental stages

**Figure 1:**
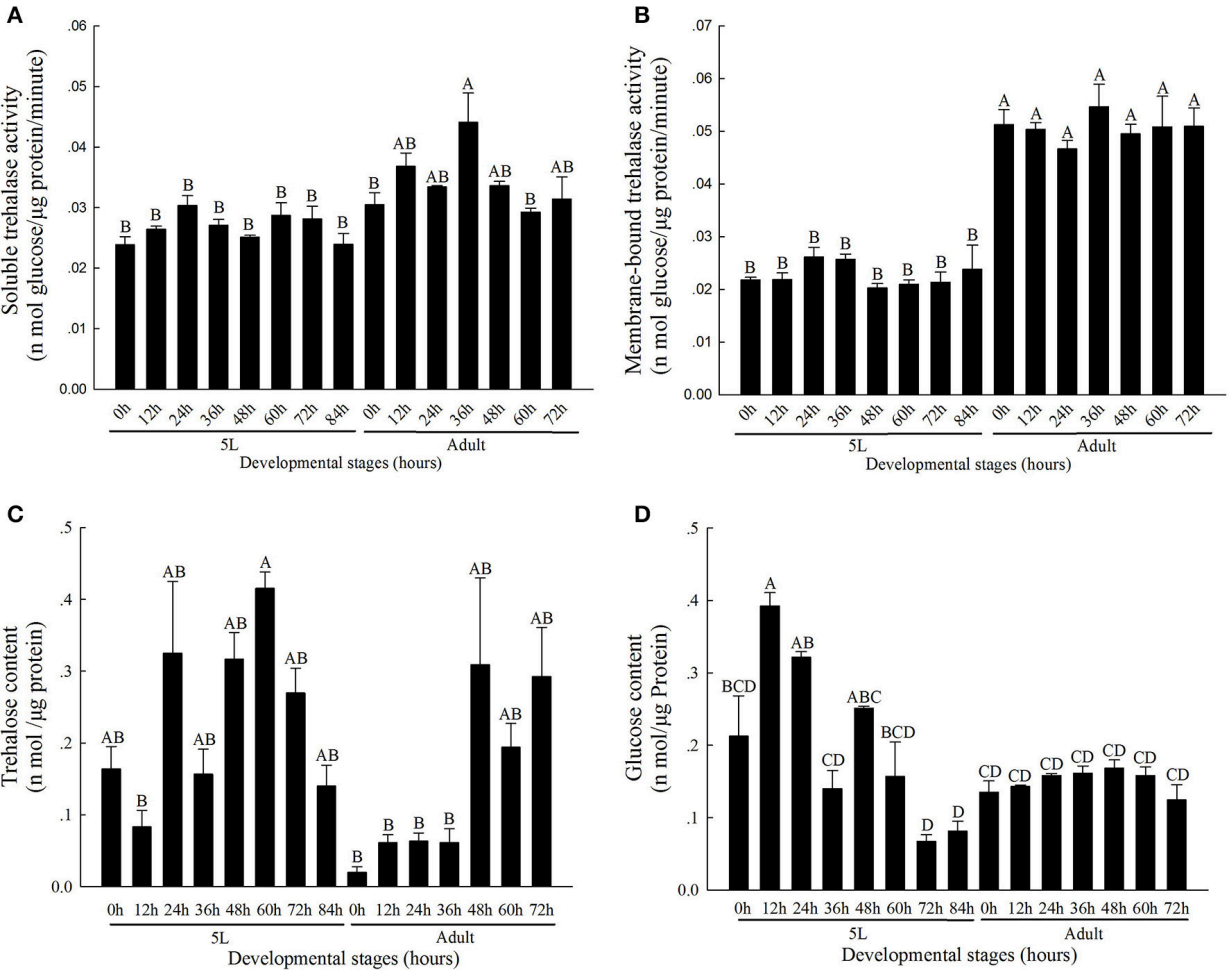
Soluble trehalase **(A)**, Membrane-bound trehalase activity **(B)**, and trehalose **(C)**, and glucose **(D)** levels in *N. lugens* at different developmental stages from 0 h in 5th instar nymph to 72 h in adults. All of *N. lugens* 5th instar nymph and adults were selected every 12 h, and insects were collected and used to detect the activities of the two kinds of trehalase isoenzymes and measure the trehalose and glucose levels. Every group had three to five replicates. The age of the brown planthoppers was defined as follows: 5L-0, 0 h fifth-instar nymph; 5L-12, 12 h fifth-instar nymph; 5L-24, 24 h fifth-instar nymph; 5L-36, 36 h fifth-instar nymph; 5L-48, 48 h fifth-instar nymph; 5L-60, 60 h fifth-instar nymph; 5L-72, 72 h fifth-instar nymph; 5L-84, 84 h fifth-instar nymph; A-0, 0 h adults; A-12, 12 h adults; A-24, 24 h adults; A-36, 36 h adults; A-48, 48 h adults; A-60, 60 h adults; and A-72, 72 h adults. (Tukey's test, α = 0.05, A>B>C>D).

The activity level of TRE1 remained relatively low during the molting process and was higher after molting (Figure 1A). The TRE1 activity increased from 0 to 24 h, decreased from 24 to 48 h, and remained relatively higher 60 and 72 h after the stage of the 5th instar nymph. In addition, TRE1 activity increased in the first stage until 36 h and then decreased from 36 to 72 h in the adult stage. TRE2 activity was lower in the 5th instar nymph and higher in adults (Figure 1B).

The contents of trehalose and glycogen changed during the molting process. The contents of these two sugars decreased from the 5th instar nymph middle stage to the molting staged and then increased. The trehalose content remained at the lowest level when molting completed at 0 h of adulthood, at 0.0200 ± 0.008 nmol μg^−1^ protein, and remained low from 12 to 36 h in the adult stages (Figure 1C); in contrast, the activities of TRE1 and TRE2 increased (Figures 1A,B). However, the glycogen content was low at 72 and 84 h in the 5th instar stage and then increased during the adult stages (Figure 1D).

### Expression of trehalase genes in different tissues

**Figure 2:**
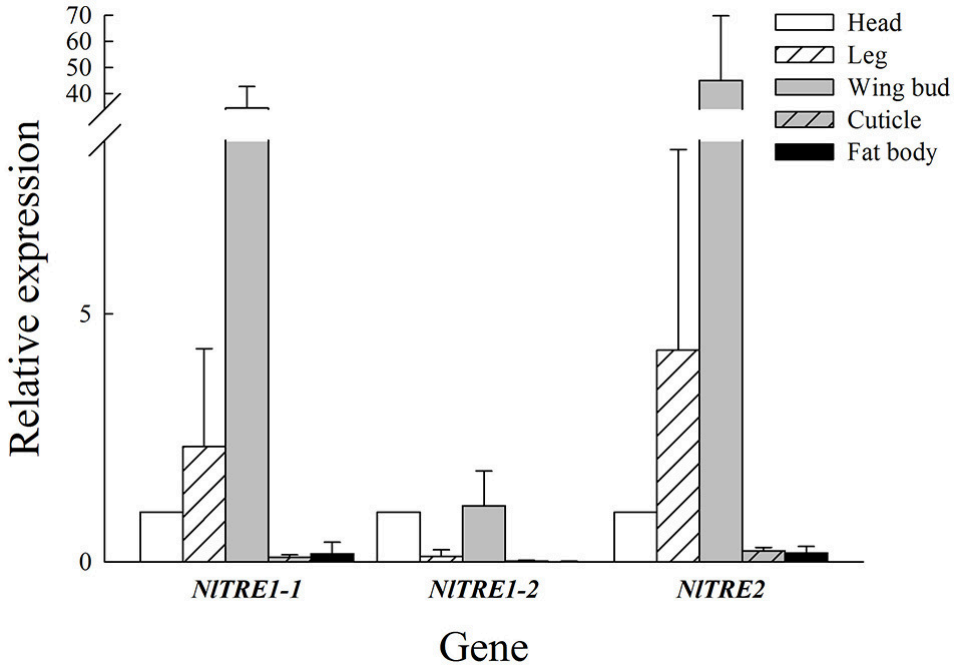
Expression of *NlTRE1-1, NlTRE1-2*, and *NlTRE2* in the five different tissues of *N. lugens* analyzed. Total RNA was extracted from head, leg, wing bud, cuticle, and fat body tissues, and the expression of both genes was measured by quantitative real-time PCR using 18S RNA as the internal control. Values are the means ± standard errors (SEs) from three independent measurements. The relative expression of each gene was determined in relation to that obtained in the head of *N. lugens* adults.

According to the qRT-PCR results, *NlTRE1-1* and *NlTRE2* had similar trends in mRNA expression but different levels of gene expression among the five tissues: head, leg, wing bud, cuticle, and fat body. As shown in Figure 2, the highest expression of both genes was registered in wing bud tissues, followed by leg and head tissues, and the lowest in cuticle and fat body tissues. The quantitative analysis of the expression of the three *NlTRE* genes in the different tissues used the expression values obtained in head tissues as the controls. The expression level of *NlTRE1-2* was relatively higher in the head and wing bud tissues than in the other tissues (Figure 2). Overall, the results showed that three *NlTRE* genes were differentially expressed across the several tissues analyzed and that their expression was significantly higher in the wing bud tissues of the BPH, followed by head tissues.

### Expression of trehalase genes in wing bud tissue and the observation of wing variation after RNA interference

**Figure 3:**
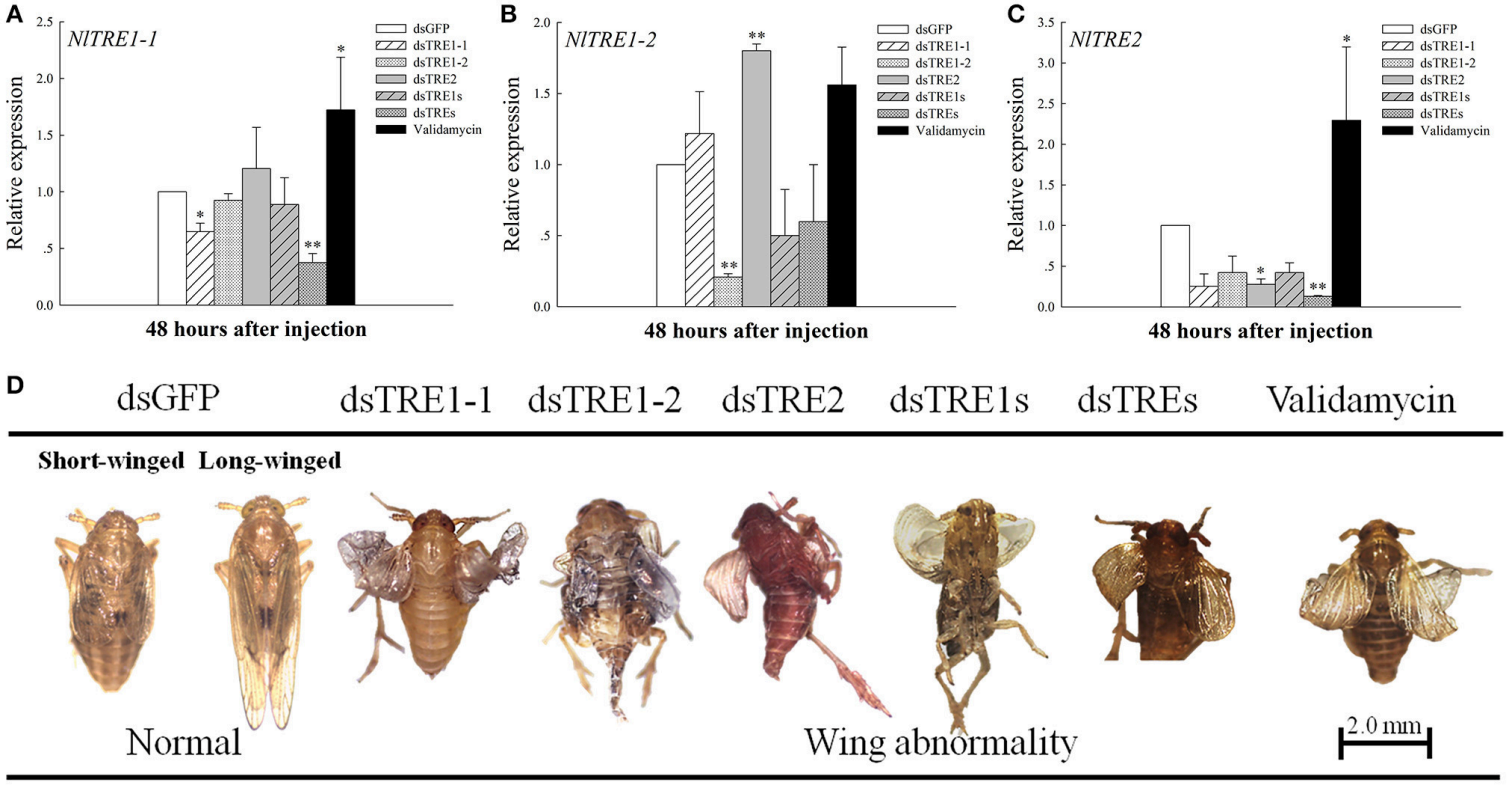
Changes in the mRNA transcript levels in wing bud and abnormal phenotypes of the three *NlTRE* genes 48 h after injection with specific RNAi constructs and validamycin in *N. lugens* nymph. **(A–C)** Represent the mRNA levels of *NlTRE1-1, NlTRE1-2*, and *NlTRE2* after the injection of RNAi targeting the *NlTRE* genes or validamycin normalized relative to the *Nl-18S* mRNA level. **(D)** Normal and different deformity phenotypes and wing curling. Last-instar nymphs were chosen as the targets for dsRNA injection. ^*^indicated significant differences at *P* < 0.05, and ^**^Indicated significant differences at *P* < 0.01.

In the previous studies, different presage of molting or wing deformities have been detected when TRE genes were knockdown by the way of RNAi or trehalase inhibitor of validamycin (Zhao et al., 2016; Tang et al., 2017). The injection of dsRNA from a single *TRE* gene could effectively inhibit the expression of the same gene at 48 h (Figure 3). An extreme decrease in *NlTRE1-1* and *NlTRE2* expression was registered 48 h after *dsTREs* injection (*P* < 0.01), which indicates that the mixture of *dsTREs* could effectively inhibit the expression of these two genes at 48 h. *NlTRE1-1* and *NlTRE2* expression levels increased significantly after validamycin injection, and the levels of *NlTRE1-2* also increased but not significantly when comparing the validamycin-injection group with the dsGFP-injection group. However, *NlTRE1-2* expression increased significantly (*P* < 0.01) 48 h after *dsTRE2* injection (Figure 3B). With the successful silencing of the three *NlTRE* genes, the insects subjected to RNAi exhibited high mortality rates and presented various abnormal phenotypes compared with the control insects. Importantly, many insects did not complete wing development and presented an abnormal wing phenotype (Figure 3D).

### Chitin synthase gene expression in wing bud tissue after knocking down three *TRE* genes

**Figure 4:**
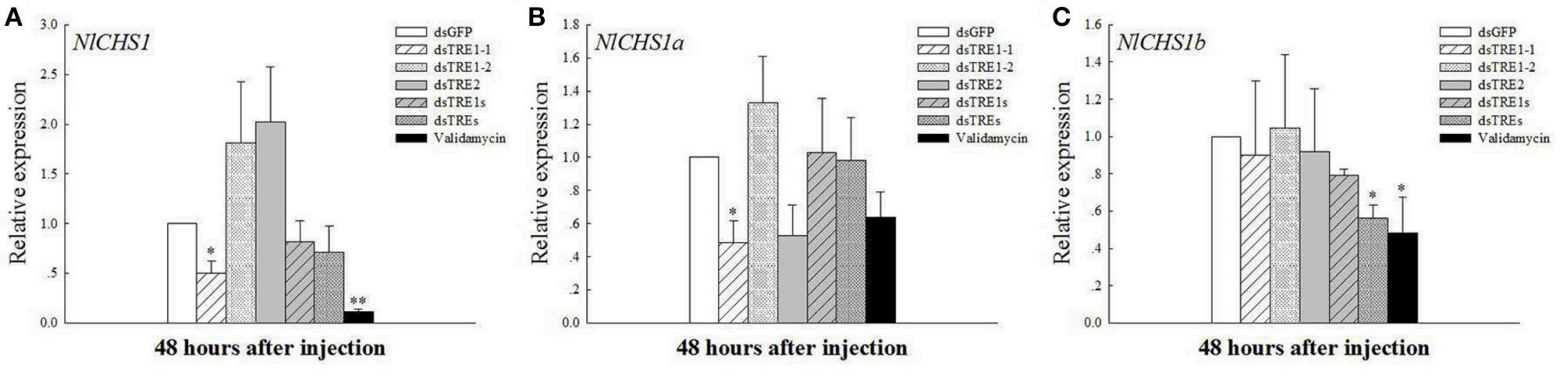
Effects of different dsTRE or validamycin solutions on the relative expression levels of chitin synthase in wing bud tissue. And CHS1 **(A)**, CHS1a **(B)**, and CHS1b **(C)** genes' expression level at 48 h normalized relative to the *NL-18S* mRNA levels, as measured by qRT-PCR. ^*^Indicated significant differences at *P* < 0.05, and ^**^Indicated significant differences at *P* < 0.01.

Chitin is the main component of the insect cuticle, trachea, and peritrophic membrane of the mid-gut (Zhu et al., 2016), and the wing bud is a part of the cuticle. The chitin content based on a mass ratio of w/w can reach up to 20% in normal insects. In addition, CHS is an essential gene in chitin biosynthesis during insect growth and development. *N. lugens* encodes only the *CHS1* gene and two variable transcripts, *CHS1a* and *CHS1b* (Wang et al., 2012). The qRT-PCR results showed that *NlCHS1* expression levels decreased significantly or extremely significantly 48 h after *dsTRE1-1* and validamycin injection and decreased after *dsTRE1s* and *dsTREs* were injected (Figure 4A). *NlCHS1a* expression decreased significantly 48 h after *dsTRE1-1* and *dsTRE2* injection (Figure 4B), while *NlCHS1a* expression also decreased significantly after *dsTREs* and validamycin injection (Figure 4C).

### Expression of *Cht* and *chitinase-*like genes after *dsTRE* and validamycin injection

**Figure 5:**
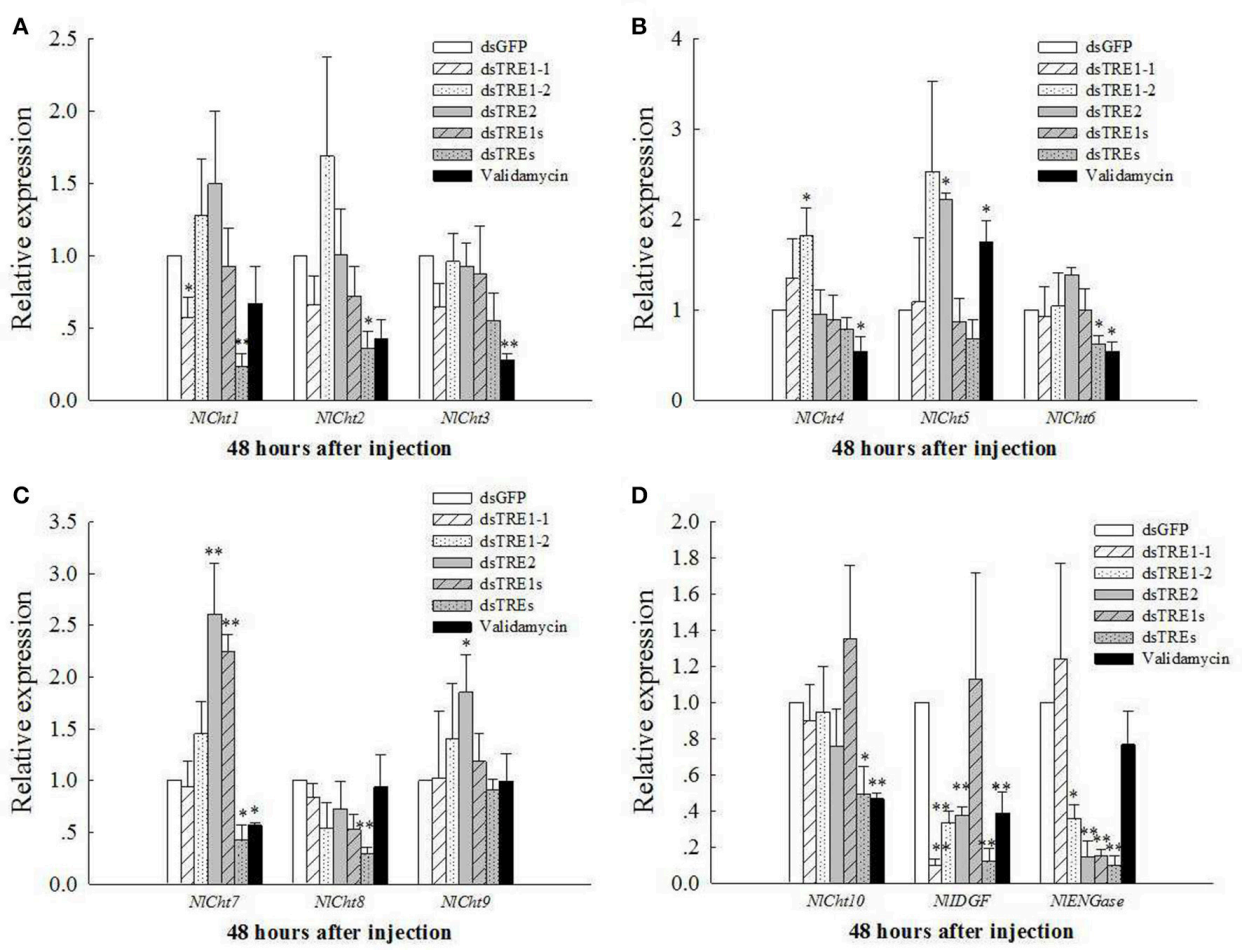
Effects of dsTREs on the chitin degradation pathway in fifth-instar nymph at 48 h. The mRNA levels of *NlCh1* to *NlCht3* **(A)**, *NlCh4* to *NlCht6* **(B)**, *NlCh7* to *NlCht9*, **(C)** *NlCh10*, one imaginal disc growth factor and one endo-β-N-acetylglucosaminidase **(D)** normalized relative to the *Nl-18S* mRNA level as measured via qRT-PCR. ^**^Indicated significant differences at *P* < 0.05, and ^**^indicated significant differences at *P* < 0.01.

The mRNA levels of *Cht* and *chitinase-like* genes, including 10 *Chts*, one *IDGF*, and one *ENGase*, after different *dsTRE* and validamycin injections were detected in wing bud tissue by qRT-PCR (Figure 5). The mRNA levels of *Cht1* and *IDGF* decreased significantly (*P* < 0.05) 48 h after *dsTRE1-1* injection (Figures 5A,D); *Cht3, Cht4, Cht6, Cht7, Cht10*, and *IDGF* expression levels decreased significantly after validamycin injection, and *Cht2, Cht6, Cht7, Cht8, Cht10, IDGF*, and *ENGase* expression levels decreased significantly or extremely significantly 48 h after injection of a mixture of *dsTREs* (Figure 5). *ENGase* expression also decreased significantly after *dsTRE1-2, dsTRE2*, and *dsTRE1s* were injected. The expression of *IDGF* also decreased extremely significantly 48 h after *dsTRE1-2* and *dsTRE2* injection (Figure 5D). *Cht4* expression increased significantly (*P* < 0.05) 48 h after *dsTRE1-2* injection (Figure 5B); *Cht5, Cht7*, and *Cht9* expression increased significantly or extremely significantly after *dsTRE2* injection; and *Cht5* and *Cht7* expression increased significantly after validamycin or *dsTRE1s* injection, respectively (Figures 5B,C).

### The expression of genes related to wing development after *dsTRE* and validamycin injection

**Figure 6:**
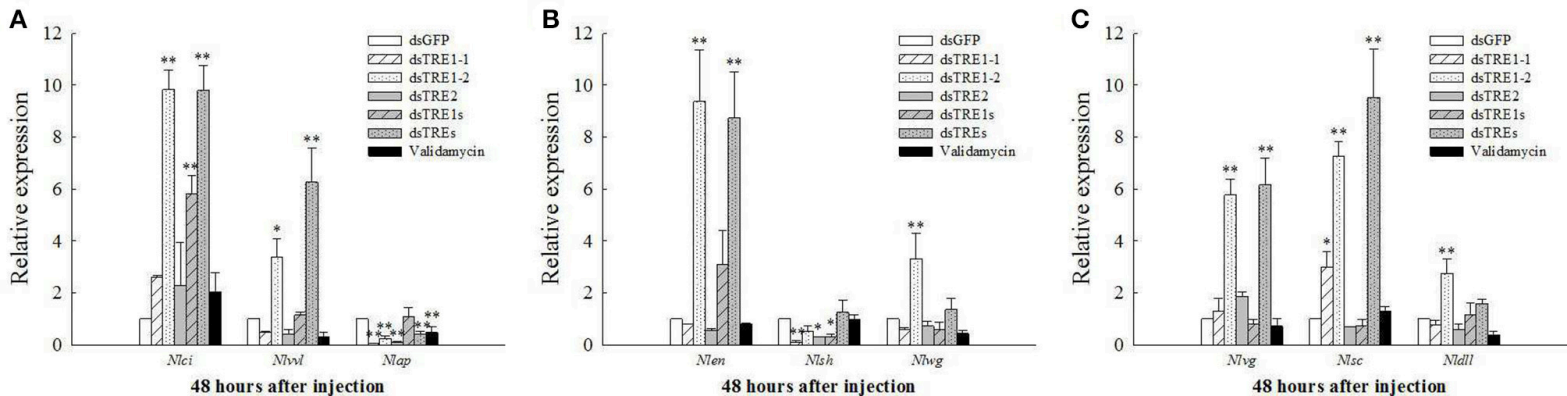
Effects of different dsTRE or validamycin solutions on the relative expression levels of wing development network genes in wing bud tissue. It is including cubitus interruptus (*CI*), ventral veins lacking (*VVL*), apterous (*AP*), **(A)** engrailed (*EN*), teashirt (*TSH*), wingless (*WG*), **(B)** vestigial (*VG*), scute (*SC*) and distal-less (*DLL*), **(C)** at 48 h normalized relative to the *Nl-18S* mRNA levels, as measured by qRT-PCR. ^*^Indicated significant differences at *P* < 0.05, and ^**^indicated significant differences at *P* < 0.01.

Moreover, the *EN, TSH, WG, DLL, VG, SC, VVL, CI*, and *AP* gene expression levels were also measured by qRT-PCR. The *VVL, CI*, and *AP* gene expression levels decreased significantly after the injection of all different concentrations of validamycin (Tang et al., 2017), while those of *VVL, AP, WG, VG*, and *DLL* also decreased after validamycin injection and did not differ significantly compared with those after dsGFP injection, except for the *AP* gene (Figure 6). The expression of the *AP* gene decreased significantly after *dsTRE1-1, dsTRE1-2, dsTRE2, dsTREs*, and validamycin injection (Figure 6A). In addition, the expression of *TSH* decreased significantly or extreme significantly after *dsTRE1-1, dsTRE2*, and *dsTRE1s* were injected (Figure 6B). However, the expression levels of these genes, including *CI, VVL, EN, VG*, and *SC*, increased significantly (*P* < 0.01 or *P* < 0.05) when *dsTRE1-2* or *dsTREs* were injected into *N. lugens* nymph (Figure 6), and *WG* and *DLL* also increased extreme significantly (*P* < 0.01) after *dsTRE1-2* was injected (Figures 6B,C).

## Discussion

Trehalose plays a key role at all developmental stages, including larvae, pupae, and adults (Becker et al., 1996; Elbein et al., 2003; Zhao et al., 2016). It not only can be degraded to glucose by two kinds of TRE but can also be synthesized by trehalose-6-phosphate synthase (TPS) and trehalose-6-phosphate phosphatase (TPP) and released in the haemolymph (Tang et al., 2016; Yang et al., 2017) or converted to glycogen by glycogen synthase (GS) (Tang et al., 2012). Trehalose and glucose contents are relatively stable and are negatively correlated in the hemolymph (Tang et al., 2010). In addition, the contents of trehalose and glucose were negatively correlated from 72 h in 5th instar nymph to 12 h in adults; this time period represents the molting process (Figures 1C,**D**). The contents of these two sugars were kept low through regulation by two kinds of TREs (Figures 1A,B). More trehalose was consumed or provided as energy during insect molting. In addition, the trehalose and glycogen contents and the activities of two TREs decreased significantly 48 h after *dsTRE1-2, dsTRE2*, and *dsTREs* were injected into *N. lugens* (Zhao et al., 2016; Zhang et al., 2017b). The glucose content also decreased 48 h after *dsTRE1* and *dsTRE2* were injected, and the trehalose content decreased 48 h after TRE2 was knocked down in *S. exigua* (Chen et al., 2010a). Insufficient trehalose during the molting process may prevent an insect from completing the chitin synthesis. Of course, more study is required to measure the amount of energy provided and ATP released when *TRE* or *CHS* and other chitin synthesis pathway genes are knocked down.

Insects can consume energy by degrading trehalose to glucose using TRE. Thus, many different insect tissues express *TRE* genes. *TRE2* is expressed in the brain, cuticle and mid gut as detected by RT-PCR (Tang et al., 2008), while *TRE1* and *TRE2* expression has been detected in all tissues, including the mid gut, Malpighian tubules, and fat body in *S. exigua* and *Apolygus lucorum* (Chen et al., 2010a; Tan et al., 2014). *TRE1* expression level is higher in the gut and lower in the integument, fat body, and embryo in *Aphis glycines* (Bansal et al., 2013). In our study, *TRE1-1* and *TRE2* expression could be detected in the head, leg, wing bud, cuticle, and fat body and was higher in head, leg, and wing bud tissues than in other tissue (Figure 2). In contrast, *TRE1-2* was expressed in the head and wing bud but not in the other three tissues (Figure 2). Leg and wing bud are components of the insect exoskeleton or cuticle, and the cuticle in our study did not **include** the leg or wing bud. The expression pattern of *TRE* genes in all kinds of tissues also differed during the different developmental stages (Tang et al., 2008; Chen et al., 2010a). The expression of other *TRE* genes may increase when one specific *TRE* gene is knocked down by RNAi (Chen et al., 2010a; Zhao et al., 2016; Zhang et al., 2017b); it is possible that similar genes have compensatory functions. The same phenomenon also observed in wing bud tissues, where *NlTRE1-2* expression increased significantly 48 h after *dsTRE2* was injected (Figure 3B), but *NlTRE2* expression decreased after *dsTRE1-1, dsTRE1-2, dsTRE2, dsTRE1s*, or *dsTREs* was injected (Figure 3C, Zhang et al., 2017b).

Insects can present all kinds of abnormal phenotypes, including molting deformity and wing deformity, during the molting process, just as they can from the larvae to pupae or from nymph to adult stages (Chen et al., 2010a; Zhao et al., 2016; Zhang et al., 2017b). In *N. lugens*, about 25% abnormal molting or wing deformity was observed when single of *dsTRE* injected, and molting deformity or wing deformity are the main abnormal phenotypes, as well as the percentage is different according to the different dsTRE gene injection groups (Zhao et al., 2016). Moreover, it exceeds 40% deformity and 60% mortality after injection 1 μg trehalase inhibitor of validamycin into each larvae of *N. lugens*. And in the different abnormal phenotypes, most of insect were shown the molting and wing deformity (Tang et al., 2017). In the same time, *CHS1a* and *CHS1b* expression decreased 48 and 72 h after single or mixed *dsTREs* was injected and *CHSA* and *CHSB* expression also decreased 48 and 72 h after *dsTRE1* or *dsTRE2* was injected in *S. exigua* (Chen et al., 2010a; Zhao et al., 2016). *N. lugens* also presented wing deformities when single or mixed *dsTRE*s was injected (Figure 3D), but *CHS1, CHS1a*, and *CHS1b* together did not decrease in the wing bud tissues. In particular, *CHS1, CHS1a*, and *CHS1b* expression increased when 48 h after *dsTRE1-2* was injected (Figure 4). In addition, some insects do not undergo normal pupation when *TREs* or *TPS* genes are knocked down, and their larval weight and survival rate decrease significantly (Shi J. F. et al., 2016). These results suggest that insect deformity may be a common result of exoskeleton and chitin synthesis blocking, which was not the only reason for wing bud development, although the chitin content of total of *N. lugens* decreased significantly (Zhang et al., 2017b).

Some previous studies have reported that blocking trehalose metabolism, which includes TPS and TRE that regulate insect's chitin synthesis and degradation, prevents insects from completing their developmental and molting processes (Chen et al., 2010b,a; Zhao et al., 2016; Yang et al., 2017). These results indicate that the chitin synthesis pathway and *chitinase* gene as expression decrease regulated by trehalose metabolism-related genes (Ge et al., 2011; Tang et al., 2017) and that the chitin content decreases significantly 48 h after TRE knockdown or TRE inhibitor injection in *N. lugens* (Zhang et al., 2017b). We found that the expression of *CHS1, CHS1a*, and *CHS1b* changed slightly and that validamycin could inhibit *CHS1* and *CHS1b* expression significantly (Figure 4). The expression of almost all *Chts* and chitinase-like genes, except *Cht5, Cht8*, and *Cht9*, as well as *Cht3, Cht4, Cht6, Cht7, Cht10*, and *IDGF*, in wing bud tissue decreased significantly 48 h after validamycin injection (Figure 5). These results show that validamycin can inhibit the activities of two kinds of TREs and lead to insect molting and wing deformities by affecting or regulating chitin synthesis and degradation in wing bud and cuticle or total tissues (Tang et al., 2017; Zhang et al., 2017b). The molting process of insects can be affected when the *chitinase* or *CHS* gene expression decreases (Zhu et al., 2008, 2016; Xi et al., 2015b), and the same kinds of deformities, including failure to break up the old cuticle during the molting process and wing deformity were found in this study. These results are similar to other observations that the expression of TPS, chitinase, TRE and CHS-or chitin synthesis pathway-related genes was knocked down. They also show that RNAi is effective for the pest management of *N. lugens* and other insects (Chen et al., 2008, 2010b,a; Zhu et al., 2008; Arakane et al., 2009; Xi et al., 2014, 2015a,b; Zhao et al., 2016; Yang et al., 2017; Zhang et al., 2017b).

Only *IDGF* and *ENGase* gene expression decreased when the *TRE* gene was knocked down, and the expression of many of the *Chts* and *chitinase-like* genes decreased in wing bud tissue 48 h after the injection of a mixture of *dsTREs* (Figure 5). When the expression of a number of wing development-related genes, including *EN, TSH, WG, DLL, VG, SC, VVL, CI, AP*, and UBX, is inhibited by RNAi or another method, insect wing abnormal phenotypes such as wing deformity (Figure 3D), small wings and curled wings are observed (Casares and Mann, 2000; Wu and Cohen, 2002; Lin et al., 2014; Xue et al., 2014; Tang et al., 2017). However, wing deformity is observed as a result of the knockdown of any *TRE* gene; wing development-related genes may be affected when different *dsTREs* are injected into wing bud tissue. We found that the *AP* or *TSH* gene' expression level decreased significantly after the injection of three single TRE dsRNAs or a mixture of *dsTRE1s* and *dsTREs* (Figures 6A,B) and conclude that *AP* or *TSH* gene silencing in wing bud tissue maybe the main reason for halted wing development or wing curling, as *AP* and *TSH* are the most important genes and play a key role during the development of the *D. melanogaster* wing (Fasano et al., 1991; Bieli et al., 2015). Dorsoventral axis formation in the *Drosophila* wing depends on the activity of the selector gene *AP* (Milán and Cohen, 2000), which was found in two studies to be a key regulatory gene involved in insect wing development (Niwa et al., 2010), and the wing blade does not develop in *AP* mutants (Diaz-Benjumea and Cohen, 1993; Williams et al., 1993; Klein et al., 1998), The *TSH* gene is required for proper formation of the trunk segments within the developing embryo (Fasano et al., 1991). *D. melanogaster* is a complete metamorphosis insect, while *N. lugens* is an incomplete metamorphosis insect which has long-wing and short-wing types (Xue et al., 2014). For these two different kinds of insects, there may be some different proteins that are implicated in the development of wing and it needs to be further studied. Nevertheless, *N. lugens* wing deformity in wing bud tissue may be due to lower expression of the *AP* and *TSH* genes when the *TRE* gene is knocked down. *TRE* can regulate the chitin metabolism pathway to control the molting process and balance the chitin content (Zhao et al., 2016; Tang et al., 2017; Zhang et al., 2017b).

At the same time, RNAi has been widely used to investigate gene function in insects and has been especially employed to silence vital genes in *N. lugens* and other insects through the suppression of gene expression by injecting dsRNA or single-stranded RNA (siRNA) (Belles, 2010; Wang et al., 2012; Kola et al., 2015; Liu et al., 2015; Joga et al., 2016). Sustained and effective RNAi expressing dsRNA in the host plant or transgene-mediated RNAi developed to decrease gene expression at the same time will be very useful for pest control (Baum et al., 2007; Mao et al., 2007; Swevers et al., 2013; Zhu, 2013; Jiang et al., 2016; Yu et al., 2016; Gillet et al., 2017). TRE has become an important target in pest management and control, and new and effective TRE inhibitor products with practical significance as pesticides are being developed (Tang et al., 2012, 2017). Therefore, there maybe two potential ways to control agriculture pests, one is development and application of effective trehalase inhibitors, and the other is plant produced sustained and effective dsTRE or dsRNA's of other important genes over time.

## Ethics statement

All applicable international, national, and/or institutional guidelines for the care and use of animals were followed.

## Author contributions

Conceived and designed the experiments: LZ, SW, MZ, and BT. Performed the experiments and analyzed the data: LZ, LQ, HY, and HW. Contributed reagents/materials/analysis tools: LZ, MZ, SW, and BT. Wrote the paper: LQ, LZ, and BT.

### Conflict of interest statement

The authors declare that the research was conducted in the absence of any commercial or financial relationships that could be construed as a potential conflict of interest.
